# Structures of CD200/CD200 Receptor Family and Implications for Topology, Regulation, and Evolution

**DOI:** 10.1016/j.str.2013.03.008

**Published:** 2013-05-07

**Authors:** Deborah Hatherley, Susan M. Lea, Steven Johnson, A. Neil Barclay

**Affiliations:** 1 Sir William Dunn School of Pathology, University of Oxford, South Parks Road, Oxford OX1 3RE, UK

## Abstract

CD200 is a widely distributed membrane glycoprotein that regulates myeloid cell activity through its interaction with an inhibitory receptor (CD200R). The interaction is of interest as a target for treating excessive inflammation and for treating leukemia. There are closely related proteins to CD200R that give activating signals making this a “paired receptor.” We report X-ray crystallography structures for the inhibitory CD200R, the activating receptor CD200RLa, and a complex between CD200R and CD200. Both CD200 and CD200R contain two Ig-like domains and interact through their NH_2_ terminal domains compatible with immunological synapse-like interactions occurring between myeloid cells and other CD200-expressing cells. The failure of the activating receptor to bind CD200 resides in subtle changes around the interface. CD200 has been acquired by herpes viruses to mimic the host interaction. CD200R has evolved rapidly presumably driven by pathogen pressure but it may also be important in homeostasis through interactions with commensal bacteria.

## Highlights

• Structure of CD200R and in complex with its ligand CD200 complex
• Structure of an activating member of the CD200 receptor family
• Mutagenesis showing specificity of CD200 paired receptor family

## Teaser

Hatherley et al. present a structural basis explaining why the cell surface protein CD200 binds to its receptor (CD200R) but not to the related activating receptor CD200RLa. The rapid evolution of the receptors was probably driven by pathogens and might play a role in mediating interaction with the microbiome.

## Introduction

CD200 (formerly termed OX2) is a widely distributed cell surface protein that interacts with a receptor (CD200R) that is highly expressed on myeloid and some lymphoid cells (Wright et al., 2000, 2003). CD200R contains tyrosine motifs that can be phosphorylated. These motifs signal through the recruitment of the adaptor DOK2, which distinguishes CD200R from almost all other inhibitory receptors, that contains immunoreceptor tyrosine-based inhibition (ITIM) motifs that provide inhibition following recruitment of phosphatases (Mihrshahi et al., 2009; Zhang et al., 2004). The broad tissue distribution of CD200 and changes in its level of expression provide a mechanism for locally regulating myeloid cell activity at appropriate sites, such as inflamed tissue (Barclay et al., 2002; Mukhopadhyay et al., 2010; Nathan and Muller, 2001). The gene for CD200 has been acquired by several viruses and in some cases has been shown to cause downregulation of myeloid activity (Foster-Cuevas et al., 2004, 2011; Rosenblum et al., 2004; Zhang et al., 2009). The CD200/CD200R homeostatic mechanism is of major interest as a target for immunomodulation both to reduce myeloid activity in inflammatory conditions and to block inhibitory signals provided by cancer cells. Inflammation is ameliorated by cross-linking the receptor with CD200-Fc fusion proteins in models of arthritis and allograft rejection (Gorczynski et al., 1999, 2001) or by antibodies in virus-induced cytokine storm (Snelgrove et al., 2008). High levels of CD200 have been associated with poor prognosis in certain cancers, suggesting that CD200 may be protecting the tumor by inhibiting myeloid cells through CD200R, and CD200 mAb are being tested to restore myeloid cell activity and help eliminate tumor cells (Kretz-Rommel et al., 2007; Moreaux et al., 2006; Tonks et al., 2007). The CD200/CD200R interaction has similarities with the CD47 interaction with the SIRP paired receptor family in that both ligands are widely distributed but receptors are restricted mainly to myeloid cells and both CD200 and CD47 are targets for leukemia therapy (Chao et al., 2012; Hatherley et al., 2008). However the inhibitory SIRPα is a conventional inhibitory receptor using ITIM motifs recruiting phosphatases (Barclay and Brown, 2006).

**Figure 1:**
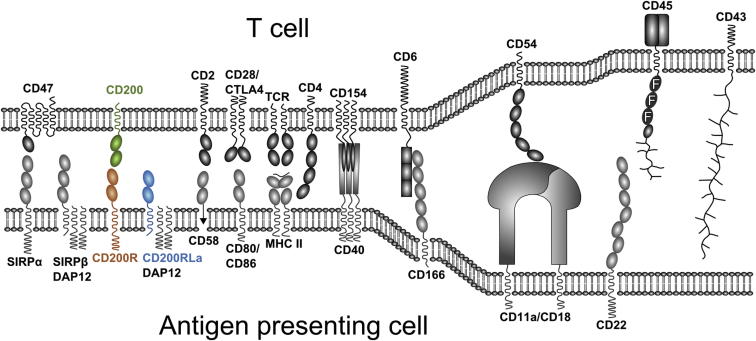
Schematic Illustrating Interactions between an Antigen-Presenting Cell and T Cell Immunogloblin domains are shown as ovals. Fibronectin domains are shown as ovals with the letter F inside. Scavenger receptor cysteine-rich domains are shown as rectangles. Tumor necrosis factor receptor (TNFR) and TNF domains are represented by long thin rectangles and long thin ovals, respectively. The mucin-like structure of CD43 and the N-terminal glycosylated region of CD45 are shown as lines.

Both CD200 and CD200R contain two Ig-like domains and a single-pass transmembrane region. They are heavily glycosylated with six potential N-linked sites in CD200 and typically six to ten in CD200R according to species (Barclay and Ward, 1982; Mallett et al., 1990; Wright et al., 2000, 2003). CD200R is a member of a “paired receptor” family as in addition to this inhibitory receptor there is one very closely related gene with activating potential in humans and several in mice (Akkaya and Barclay, 2010; Wright et al., 2003). These were termed CD200RL for receptor-like but have also been termed CD200R1, CD200R2, etc. (Gorczynski et al., 2004; Voehringer et al., 2004). What is intriguing about several paired receptors is that their extracellular regions typically have about 90% amino acid sequence identity but there is a major difference in ligand binding between the activating and inhibitory members. The activating CD200RL proteins give negligible binding to CD200 so that the change of function from inhibitory to activatory is associated with a loss of ability to interact with host ligand (Hatherley et al., 2005). One theory is that this evolution is being driven by pathogens targeting the inhibitory receptor (Akkaya and Barclay, 2013; Barclay and Hatherley, 2008). The interaction between CD200 and CD200R involves their NH_2_ terminal domains and hence is predicted to span about four Ig-like domains typical of interactions in immunological synapses (Figure 1). Because CD200 is expressed on nonlymphoid cells as well as leukocytes, it seems likely that synapse-like interactions may be widely utilized (Wright et al., 2000).

We describe the X-ray crystallographic analysis of the extracellular regions of the mouse CD200R, one of the activating receptors, CD200RLa, and a complex between CD200R and its ligand CD200. We discuss the implications for paired receptor specificity, the evolution of these receptors and pathogen pressure, and the implications of the topology of the interaction in constraining the engagement of the receptor.

## Results and Discussion

### Production of Recombinant Proteins for X-ray Crystallographic Analysis

To provide homogenous protein for X-ray crystallography, the extracellular regions consisting of two Ig-like domains of CD200, CD200R, and CD200R-like proteins were expressed in CHO cells that were defective in glycosylation so that the N-linked glycosylation sites did not mature beyond the high mannose state (Davis et al., 1993; Stanley, 1989), making it possible to obtain more homogenous protein by endoglycosidase H treatment. Glycoforms of CD200 and CD200R resistant to endoglycosidase H were removed by passing the recombinant proteins through a concanavalin A affinity column that preferentially binds high mannose structures, allowing recombinant protein deglycosylated back to N-acetyl-glucosamine (NAG) to pass through the column (Figure S1 available online).

### Crystallization and Structure Determination

**Figure 2:**
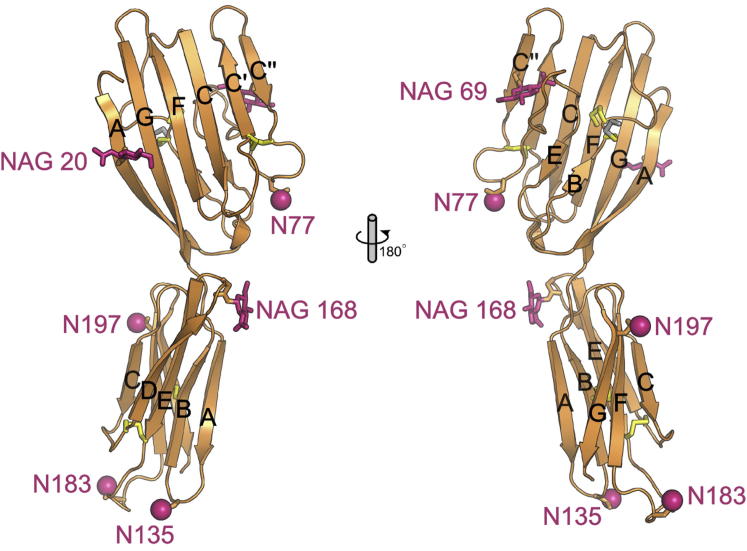
Structural Characteristics of Mouse CD200R. The mouse CD200R structure is shown as an orange cartoon. Beta strands of each Ig-like domain are labeled A-G. Observed N-acetylglucosamine (NAG) moieties (N20, N69 and N168) are shown as pink sticks and other potential N-linked glycosylation sites are represented by pink spheres N77, N135, N183 and N197. Disulfide bonds are shown as yellow sticks. Cys 34 is shown forming a disulfide bond with a free Cys (gray stick). CD200R is rotated 180° in right hand panel. See also Figure S2.

**Figure 3:**
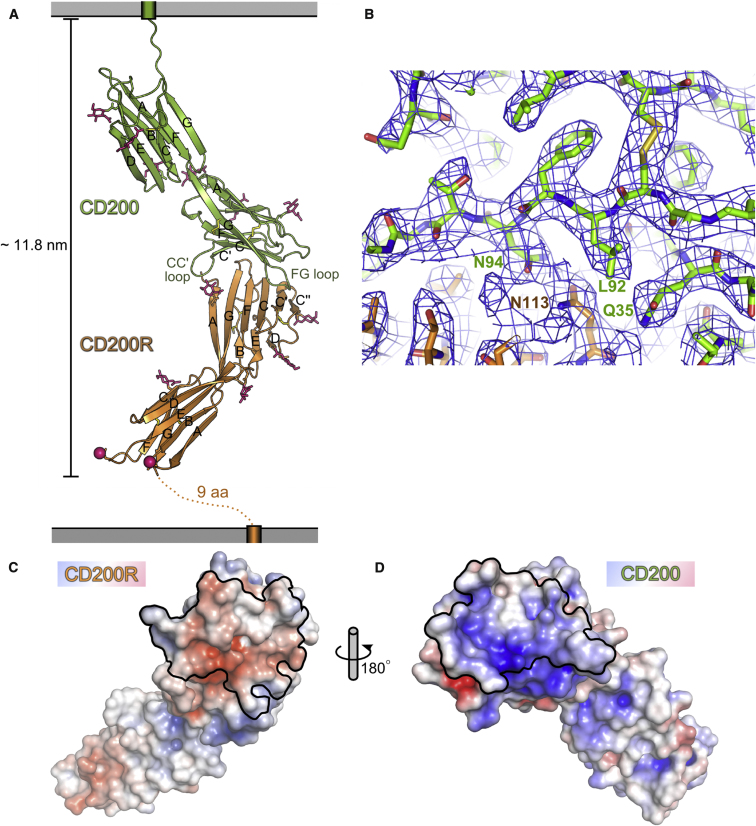
Structure of the CD200/CD200R Complex (A) The complex is shown with CD200 in green and CD200R in orange. Beta strands of each Ig-like domain are labeled A–G. Observed NAG moieties are shown as pink sticks and potential N-linked glycosylation sites are represented by pink spheres. Disulfide bonds are represented as yellow sticks. (B) Representative electron density showing CD200 (green carbon atoms) and CD200R (orange carbon atoms) at the interaction interface. The final refined model is shown in 2Fo-Fc electron density (1 σ) calculated at the end of refinement. (C and D) Open book representation of the CD200 / CD200R interface. The surface of CD200R as viewed by CD200 (C). The molecular surface of CD200R is shown colored by electrostatic potential with the residues at the interaction interface bordered by a black line. The surface of CD200 as viewed by CD200R (D). The molecular surface of CD200 is shown, colored by electrostatic potential with the residues at the interaction interface bordered by a black line. See also Figure S3.

Crystals were obtained for the extracellular regions of CD200RLa, CD200R alone, and CD200R in complex with CD200 and native diffraction data were recorded to 2.6, 2.1, and 3.2 Å, respectively (Table 1). The structure of CD200RLa was solved after phase determination using crystals soaked in gadolinium chloride solution because molecular replacement (MR) phasing was unsuccessful. The structure of CD200R was solved by molecular replacement using the coordinates of CD200RLa as a search model (Figure 2). The coordinates of CD200R were used as a MR search model for the CD200R/CD200 complex and CD200 was built in a map calculated using partial MR phases. Details of the crystallographic data collection and refinement are given in Tables 1 and 2, and the structures are given in Figures 2 and 3.

**Table 1:** Data Collection and Refinement Statistics

	CD200RLa (native)	CD200RLa (gadolinium)	CD200R	CD200/CD200R Complex
**Data Collection Statistics**				

Beamline	ID23-1 (ERSF)	I03 (diamond)	I04 (diamond)	I04 (diamond)
Wavelength (Å)	0.9762	1.7112	0.9795	0.9840
Resolution limits (Å)^a^	168.02–2.5 (2.63-2.5)	137.05–3.5 (3.69-3.5)	38.84–2.08 (2.13-2.08)	86.04–3.22 (3.31-3.22)
Space group	*P6*_*5*_*22*	*P6*_*5*_*22*	*P*4_1_ 2_1_ 2	*P*4_3_ 2_1_ 2
Unit cell dimensions (Å, °)	157.76, 157.76, 167.91 90, 90, 120	158.25, 158.25, 167.5 90, 90, 120	51.97, 51.97, 175.42	128.17, 128.17, 115.08
			90, 90, 90	90, 90, 90
Total number of observations	151,989	93,847	116,395	101,278
Unique reflections	42,242	16,156	15,423	16,034
Multiplicity^a^	3.6 (3.5)	5.8 (5.9)	7.5(7.3)	6.3 (6.6)
Completeness (%)^a^	98.4 (99.7)	99.8 (99.8)	99.8 (99.5)	99.6 (99.5)
*Iσ* (*I*)^a^	13.0 (2.2)	10.9 (5.1)	16.8 (3.1)	10.2 (2.9)
*R*_merge_ (%)^a^^,^^b^	6.9 (49.4)	15.4 (39.0)	7.8 (73.8)	12.0 (68.3)
*R*_anom_ (%)^a^^,^^c^		9.0 (15.9)		
Processing programs	MOSFLM/SCALA	XIA2/SCALA	XIA2	XIA2

**Refinement Statistics**				

Resolution limits (Å)	15.0–2.5 (2.56–2.5)		15.0–2.08	86.04–3.22
Number of reflections in working set	39,601		14,424	14,465
Number of reflections in test set	2,115		758	819
*R*-factor of working set a^,^^d^	0.171 (0.248)		0.198 (0.236)	0.199 (0.313)
*R*_free_^a^^,^^e^	0.205 (0.291)		0.232 (0.270)	0.251 (0.360)
Number of atoms (protein/carbohydrate/water/other^f^)	4,328/210/625/53		1,464/42/148/11	3,035/154/27/4
Residues in Ramachandran-favored region (%)	98.4		97.3	94.1
Ramachandran outliers (%)	0.0		0.0	0.0
Rmsd bond lengths (Å)	0.010		0.0100	0.007
Rmsd bond angles (°)	1.23		1.32	1.40
Average *B* factors (Å^2^) (protein/carbohydrate/water/other^f^)	45/83/55/107		39/42/48/55	93/122/63/68

a Numbers in parentheses refer to the appropriate outer shell.

b *R*_merge_ = 100 x (Σ_hkl_ Σ_i_|*I(hkl;i)* − < *I(hkl)* > |/Σ_hkl_ Σ_i_*I(hkl;i))*, where *I(hkl;i)* is the intensity of an individual measurement of a reflection and < *I(hkl)* > is the average intensity of that reflection.

c *R*_anom_ = 100 x Σ |Mn(I+) − Mn(I−)|/Σ (Mn(I+) + Mn(I−)) where R_anom_ is the ratio between the mean intensity difference between Bijvoet pairs (|Mn(I+) − Mn(I−)|) and the mean intensity of Bijvoet pairs (Mn(I+) + Mn(I−).

d *R*_factor_ = (Σ_hkl_||*F*_obs_| − |*F*_calc_||/Σ_hkl_ |*F*_obs_|), where |*F*_obs_| and |*F*_calc_| are the observed and calculated structure facture amplitudes.

e *R*_free_ equals the *R*-factor of test set (5% of the data removed prior to refinement).

f Other atoms include polyethylene glycol, sulfate, glycerol, acetate.

**Table 2:** Experimental Phasing Statistics for CD200RLa

Phasing Power	SIR		SAD
Resolution Bin	Acentric	Centric	Acentric
78.8–11.08 Å	5.174	3.221	1.508
11.08–7.87 Å	3.400	2.192	1.172
7.87–6.44 Å	2.213	1.487	0.769
6.44–5.58 Å	1.457	0.990	0.505
Overall	0.918	1.064	0.359
	FOM Acentric^a^	FOM Centric^a^	
78.8–11.08 Å	0.83238	0.71582	
11.08–7.87 Å	0.71049	0.65144	
7.87–6.44 Å	0.55196	0.54371	
Overall	0.07967	0.15751	

a The overall figure of merit (FOM) is before density modification. After solvent flattening, the FOM is 0.894. Only resolution bins with phasing power >1 or FOM >0.5 are shown.

### Structural Characteristics of CD200R and CD200RLa

**Figure 4:**
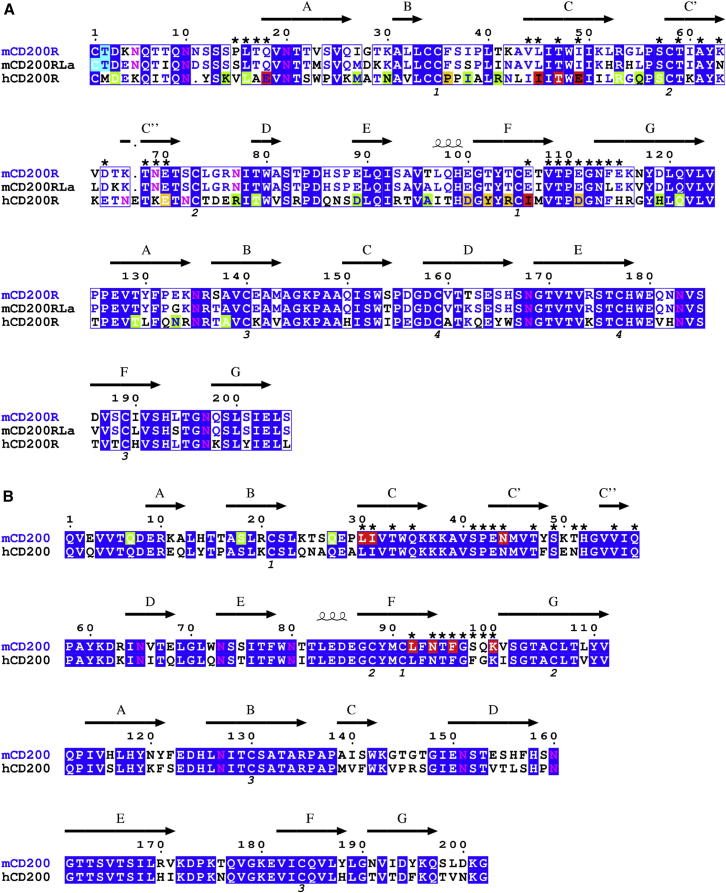
Sequence Alignment of the Extracellular Regions of the CD200R Family and CD200 The sequences for mouse CD200R (NP_067300), mouse CD200RLa (NP_997127) and human CD200R (NP_740750) are shown in (A) and the sequences for mouse and human CD200 (accession numbers NP_034948 and EAW79672 respectively) are shown in (B). Amino acid secondary structure is based on the mouse CD200R and CD200 structures. Residues identical between the sequences are highlighted in blue. NH_2_-terminal residues determined by protein sequencing are highlighted in cyan; CD200RLa consisted of a mixture of two different NH_2_ termini differing by one amino acid starting with either Cys or Thr. The NH_2_ termini of recombinant human and mouse CD200 was not detected presumably due to the presence of cyclized glutamine (pyroglutamic acid) as predicted from peptide analysis of rat CD200 (Clark et al., 1985). Potential N-linked glycosylation sites are shown in pink. Cys residues forming disulfide bonds are numbered below the alignment. Residues at the CD200/CD200R interface are denoted by asterisks. Cys residues forming disulfide bonds are numbered below the alignment. Residues at the mouse CD200/CD200R interface are denoted by asterisks. Single point mutations of human CD200R (Hatherley and Barclay, 2004) and mouse CD200 are highlighted according to their ability to bind human CD200 and mouse CD200R respectively; red denotes mutation results in < 25% wild-type binding, yellow 25%–75%, and green > 75% binding.

CD200R and CD200RLa contain typical Ig-like domains consisting of an NH_2_-terminal V-like domain followed by a smaller C2-like domain. In both molecules, the A strand of the V-like domain is on the extensive GFCC′C″ face as is commonly found in Ig-like domains (Barclay, 2003) and there is a short alpha helix in the EF loop of the V-like domain. NH_2_-terminal amino acid sequencing confirmed the presence of an usually long NH_2_-terminal region prior to the predicted beginning of the IgSF V-like domains of CD200R and CD200RLa (Figure 4). The first NH_2_-terminal residue for which electron density was observed is P15 in CD200R and Q18 for CD200RLa. The absence of electron density for the potentially glycosylated NH_2_-terminal region suggests this region is flexible and able to adopt different conformations.

The structure of CD200RLa is very similar to that of CD200R, as expected due to their high amino acid sequence identity in their extracellular regions (86%). There are significant differences in the loops (BC, CC′, and DE) of the V-like domain, but this is probably a consequence of their involvement in forming crystal contacts in the CD200R crystal. Superposition of the V-like domains of CD200R and CD200RLa reflects these differences with an average root mean square deviation (rmsd) of 2.05 Å over 106 Cα atoms whereas the C2-like domains superimpose with an rmsd of 0.6 Å over 80 Cα atoms.

Both CD200R and the CD200RLa contain disulfide bonds in addition to the canonical disulfide of most Ig-like domains that links beta strands B and F in each domain, which are largely conserved in mammals. Intrasheet disulfides are found between beta strand C′ and the C″D loop in the V-like domain and between beta strands D and E in the C2-like domain. Interestingly, the V-like domains of CD200R and CD200RLa have an exposed Cys (C34) at the end of beta strand B to which a short portion of continuous electron density was observed, and in which a Cys (forming a disulfide bond with C34) was modeled (Figure S2). This extra density was observed in the structures of CD200R alone, CD200R in complex with CD200, and in all three independent molecules of CD200RLa present in the asymmetric unit. Because the crystallization conditions differ for each protein, it is unlikely that the free Cys was modified by a component of the crystallization solution. Presumably CD200R and CD200RLa picked up Cys from the CHO cell medium during expression. The roles of most of these extra disulfides are presumably structural; however, other functional roles cannot be excluded (Metcalfe et al., 2011).

### Structural Characteristics of CD200

The structure of CD200 was solved in complex with CD200R and contains an NH_2_-terminal Ig V-like domain followed by a smaller C2-like domain (Figure 3). The topology is very similar to that of CD200R including the short alpha helix in the EF loop. The CD200 C2-like domain is characterized by extremely high B factors (130Å^2^) despite packing against another CD200 molecule. In addition to a disulfide bond between beta strands B and F in both IgSF domains of CD200, there is an exposed disulfide bond between beta strands F and G in the V-like domain. This disulfide bond is conserved in CD200 orthologs (including viral ones discussed subsequently), but disulfides in this position are unusual in Ig-like domains. NAG moieties were modeled at all six of the N-linked glycosylation sites (N65, N73, N80, N127, N151, and N160) of CD200.

### Structure of the CD200/CD200R Complex

The structure of the CD200/CD200R complex shows near orthogonal binding of the V-like domains of each molecule, whereby the CD200 FCC′C″ face and CC′ and FG loops form a concave surface that embraces the relatively flat AGFCC′C″ face and FG loop of CD200R (Figures 3A and 3B). Five NAG moieties were modeled for the bound CD200R at the same Asn as NAGs were modeled for CD200RLa. Despite the high content of carbohydrate in both CD200 and CD200R, it is not involved in the interaction, with N-linked glycosylation of both CD200 and CD200R distant from the interface with the exception of N20 of CD200R, which lies at the periphery of the interaction interface. The CD200/CD200R interaction buries a total of 1768 Å^2^ of solvent-accessible area (901 Å^2^ from CD200 and 868 Å^2^ from CD200R) and shows high shape complementarity with a shape correlation score (SC) of 0.69. The interaction interface also shows charge complementarity with acidic patches on CD200R complementing basic patches on CD200 (Figures 3C and 3D) where a network of polar interactions is clustered. Hydrogen bonds are formed between residues (including water-mediated hydrogen bonds) in the CD200R AGFCC′ beta strands (T17, E115, F114, N113, E106, T47, T59), FG loop (P110, E111, N113) and the CD200 FCC′ beta strands (T33, Q35, N44, T47, N94), and CC′ (S41, P42), C’’D (Q57) and the FG loops (T95, G97, S98, Q99, K100). There are two salt bridges with K100 (FG loop) of CD200-forming salt bridges with E106 (beta strand F) and E115 (beta strand G) of CD200R. Hydrophobic interactions are mediated between residues across the top of the FCC′C″ face of CD200R and residues in the C strand and FG loop of CD200. In particular F96 (in the FG loop) of CD200 mediates numerous hydrophobic interactions where the phenyl ring sits in a hydrophobic pocket formed by residues in the CD200R CC′C″ beta sheet (L45, A61, Y62, K63, T68, N69, and E70).

Superposition of the V-like domains (residues 17–126) of CD200R alone and in complex with CD200 supports the earlier suggestion that the NH_2_-terminal region of CD200R is flexible; a rotation of the main chain around residue 17 results in residues 15-16 moving from the front GFC face (which would impede binding to CD200) to the back BED face, exposing the CD200 binding face (Figure S3). Other than the NH_2_ terminus, there are no significant changes to the interacting face of CD200R upon binding. Conformational differences between the bound and unbound CD200R are seen in the BC loop (residues 37–41), DE loop (residues 82–87), and to a lesser extent in the EF loop (residues 93–94). These loops are not involved in the interaction with CD200, and these differences are due to the different packing of the molecules within the crystals and also reflect the plasticity of these loops.

### Validation of the CD200/CD200R Interface

**Figure 5:**
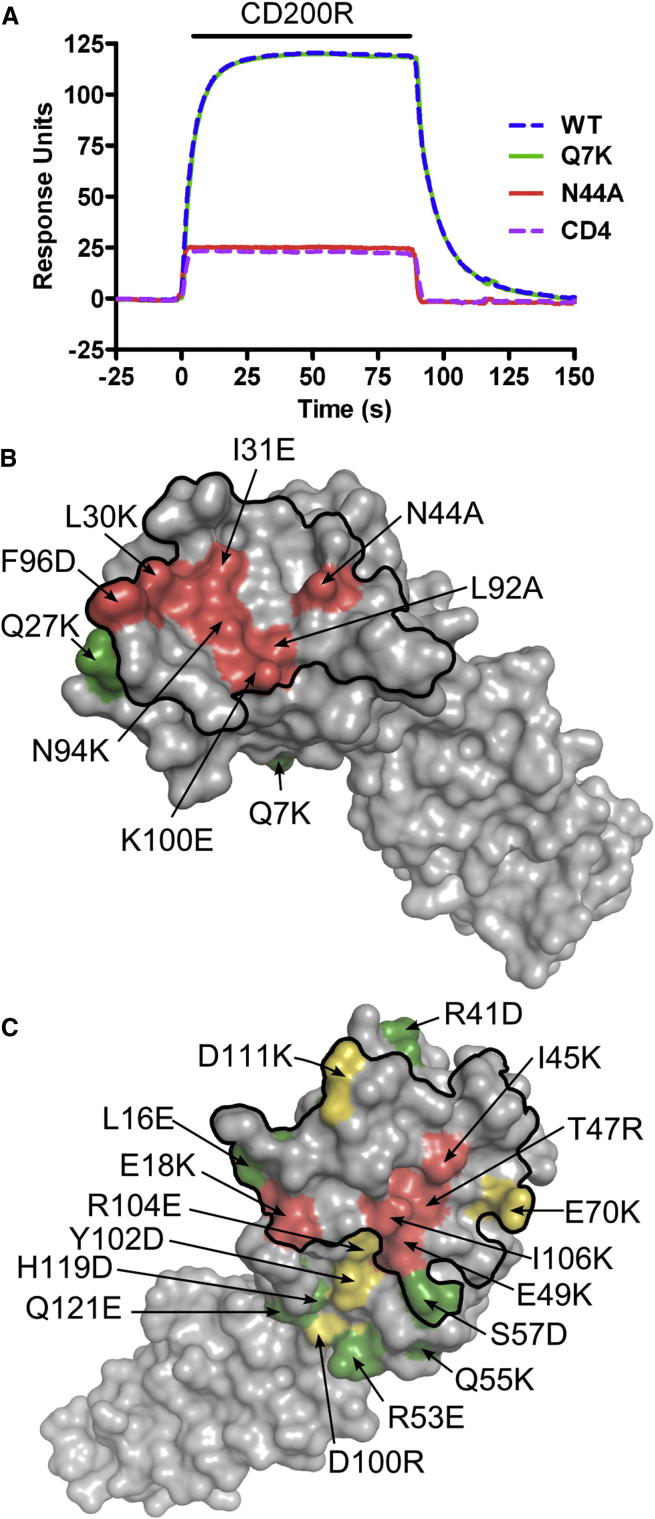
Single Point Mutations in CD200 at the CD200/CD200R Interface Prevent CD200R Binding (A) Surface plasmon resonance experiment shows immobilized CD200 mutant Q7K (green line) binds soluble CD200R similarly to wild-type (WT) CD200 (blue line), whereas CD200 mutant N44A (red line) and the negative control CD4 (pink dashed line) do not bind CD200R. Injection of soluble CD200R over the immobilized proteins is represented by the solid black bar. (B) Single point mutation of CD200 at the crystallographic CD200/CD200R interface prevents the interaction. The molecular surface of CD200 is shown in the same orientation as in Figure 3D. Single point mutations of mouse CD200 are colored according to their ability to bind mouse CD200R. Residues colored red denote mutation results in < 25% wild-type binding and green > 75% binding. (C) Mapping of single point mutations of human CD200R (Hatherley and Barclay, 2004) onto the mouse CD200R structure delineates the CD200 binding site to the AGFCC′C″ face. Residues colored red denotes mutation resulting in < 25% wild-type binding, yellow 25%–75% and green > 75% binding. (B) and (C) are in the same orientation as Figures 3D and 3C, respectively. See also Table S1.

While some key residues in the CD200/CD200R interaction had been identified in CD200R previously by mutagenesis there were few data for CD200. Single point mutants of mouse CD200 containing a CD4 tag at the COOH terminus were expressed in 293T cells and immobilized onto a BIAcore 3000 CM5 chip precoated with a CD4 mAb. The ability of each CD200 mutant to bind CD200R was assessed by passing over soluble CD200R at 0.5 μM (the K_D_ of the interaction at 25°C) and comparing the binding responses with wild-type CD200. A typical BIAcore experiment is shown in Figure 5A for wild-type CD200, mutant CD200 proteins Q7K and N44A, and a negative control protein CD4 (domains three and four). N44 of CD200 is in beta strand C′, mediating hydrogen bonding with N113 of CD200R, and mutation of N44 to alanine destroys this interaction (Figure 5A). Residue 7 (Q7) of CD200 is not involved in the interaction with CD200R and is located in beta strand A; hence mutation of this residue to lysine (Q7K) did not affect binding to CD200R. Mutation of other CD200 residues (L30, I31, L92, N94, and F96) at the CD200R binding interface also effectively destroyed ligand binding by disrupting either polar or hydrophobic interactions whereas mutations of residues outside the binding interface retained binding ability (Figure 5B; Table S1). CD200 mutant Q27K gave 150% wild-type binding response. Although Q27 does not make contact with CD200R, it is at the periphery of the interface and mutation to lysine may allow salt bridge formation with E70 of CD200R. All mutants were expressed well and reacted with the CD200 mAb OX90, which recognizes a conformationally sensitive epitope (unpublished observations).

The ligand binding domains of mouse and human CD200R share 51% amino acid sequence identity (residues 1–123; Figure 4) and human CD200 binds to mouse CD200R with a similar affinity as mouse CD200 (K_D_s of 7 and 4 μM at 37°C, respectively) (Foster-Cuevas et al., 2004; Hatherley et al., 2005). Hence the interaction is likely to be similar for both species allowing data from a previous mutagenesis study of the human proteins (Hatherley and Barclay, 2004) to be mapped onto the mouse CD200/CD200R complex. All mutated residues in human CD200R that gave less than 25% binding to CD200 as compared to wild-type protein are located at the mouse CD200/CD200R interaction interface (Figure 5C) and mutations that had an intermediate effect (25%–75%) are at the periphery of the interaction interface.

### Why Doesn’t Mouse CD200RLa Bind CD200?

**Figure 6:**
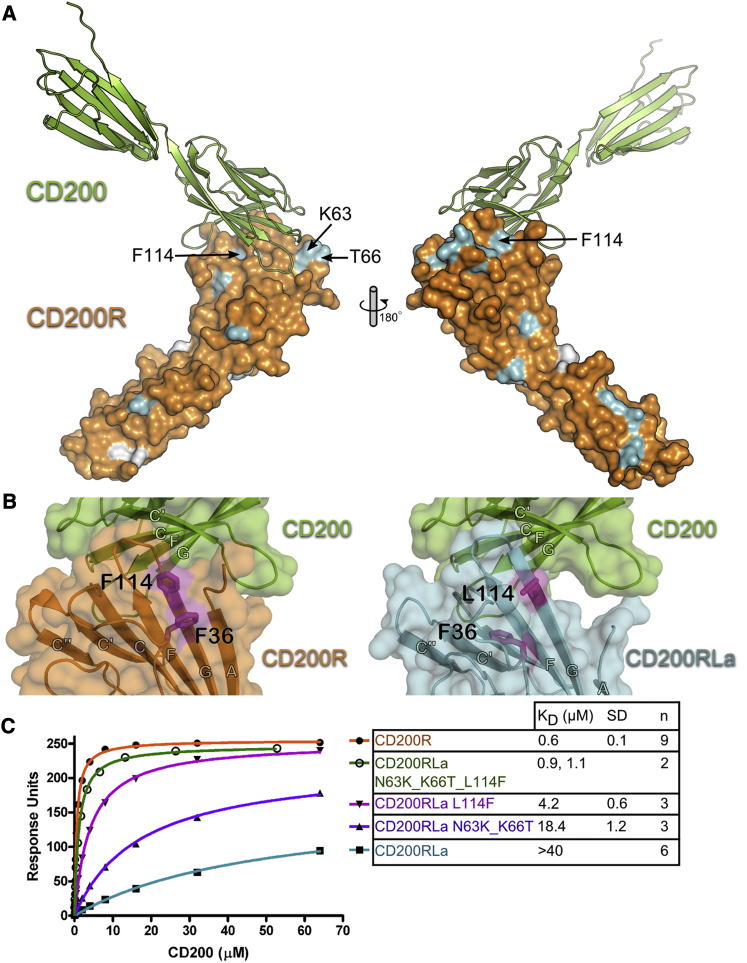
The Inability of CD200RLa to Bind CD200 (A) The structure of CD200 (green cartoon) in complex with CD200R (orange surface) is shown. Residues differing between CD200La and CD200R are colored cyan. Additional residues of CD200RLa that differ from the CD200R sequence crystallized but that are found in other CD200R variants (from Ensembl database, http://www.ensembl.org) are colored white. Three residues of CD200R that differ from CD200RLa that are at or peripheral to the CD200/CD200R interface are labeled. (B) Left panel: transparent surface and cartoon representation of the CD200 (green) in complex with CD200R (orange); F36 and F114 are shown as sticks and colored purple. Right panel: virtual complex between CD200RLa (cyan) and CD200 (green); F36 and L114 are colored purple and the loss of the N-terminal strand from the interface is highlighted. (C) Surface plasmon resonance experiments show that mutation of three CD200RLa residues (N63, K66 and L114F) is required to gain CD200 binding (∼60% of wild-type). Recombinant CD200R, wild-type CD200RLa, and mutant CD200RLa were immobilized on a BIAcore chip and increasing concentrations of CD200 were injected over the proteins. A representative experiment is shown. K_D_s were calculated by nonlinear curve fitting. The number (n) of times the experiment was repeated and the standard deviation (SD) of the K_D_s calculated are given. For differences between the receptors, see alignment in Figure S4.

Despite CD200RLa sharing a high amino acid sequence identity with CD200R in the V-like domain (84% identity in residues 1–123), it gives minimal binding to CD200 (50-fold lower affinity; Wright et al., 2003). Comparison of the CD200R and CD200RLa sequences does not show simple changes in the contact residues that might explain why the activating receptor CD200RLa does not bind CD200, implying indirect effects. To investigate this, the structure of the V-like domain of CD200RLa was superimposed onto CD200R in complex with CD200. CD200RLa and CD200R differ by 20 amino acids in the V-like domain but genome analysis shows that three of these are found as natural variants in CD200R, indicating 17 definitive differences. The first four differences between the two molecules are in the NH_2_-terminal region (residues 1–15), which is not seen in the structure of CD200RLa. Many of the amino acid differences are distant or peripheral (residues 23, 28, 29, 38, 41, 42, 52, 54, and 117) to the interface (Figure 6A). Residues 63 and 66 in the C′C″ loop differ between CD200R and CD200RLa and are at the periphery of the interaction with K63, making contact with F96 and L30 of CD200. Although residue 66 is not directly involved in the interaction, a difference at this position may adversely affect positioning of residue 63. Single point mutation of either residues 63 or 66 of CD200RLa to the equivalent residue in CD200R (N63K and K66T, respectively) does not result in CD200 binding. However the CD200RLa double mutant N63K K66T does bind CD200, although with an affinity (K_D_ = 18.4 μM) about 30 times weaker than that with CD200R (Figure 6B). Residue 114 in beta strand G is Phe in CD200R and makes contact with N44 of CD200. However, it also stacks against F36 in CD200R and together these stacked aromatic residues are involved in hydrophobic interactions with the NH_2_-terminal strand of CD200R, helping to stabilize the CD200 binding conformation of residues 15–20 of CD200R (Figure 6B). Substitution of F114 for Leu in CD200RLa seems to cause the side chain of F36 to flip in the opposite direction into the core of the domain. This in turn leads to the destabilization of the NH_2_ region of CD200RLa and therefore disruption of the CD200 binding interface (Figure 6B). Mutation of L114 of CD200RLa to Phe (L114F) results in binding to CD200 with a K_D_ of 4.2 μM, about seven times weaker than the CD200/CD200R interaction (Figure 6C). Thus both regions are important in explaining why the CD200RLa does not bind CD200 and this argument was strengthened by the analysis of a CD200RLa triple mutant combining both regions (N63K, K66T, and L114F) that binds CD200 with a similar affinity as CD200R (Figure 6C).

The three residues that are critical for CD200RLa not binding CD200 are not conserved in the other activating receptors, indicating that these fail to bind CD200 for different reasons (Figure S4). This resembles the situation seen in the SIRP family, where two activating alleles of SIRPβ use different mechanisms not to bind ligand (Hatherley et al., 2008). In addition, the CD200RLb differs greatly from the other activating receptors with only 32% amino acid identity with CD200R in the V-like domain (residues 1–124) and many residues could be important in its failure to bind CD200.

### Topology of the CD200/CD200R Interaction

**Figure 7:**
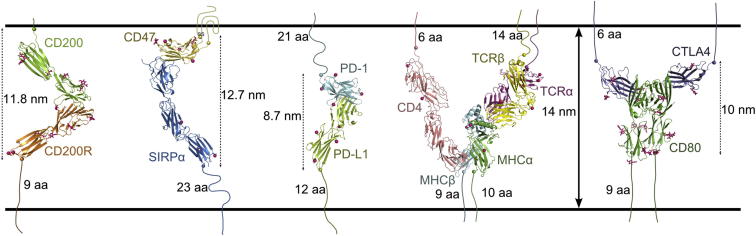
Topology of the CD200/CD200R Interaction at the Cell Surface The dimensions of the CD200/CD200R interaction are compared to other interactions thought to occur in immunologic synapses. A model of the CD47/SIRPα interaction was generated by superimposing the crystal structure of the full extracellular domain of SIRPα (Protein Data Bank ID [PDB] code 2wng) onto the crystal structure of the V-like domain of SIRPα in complex with CD47 (PDB code 2jjs). Cartoons for the crystal structures of PD-1/PD-L1 (PBD code 3bik), CD4/TCR/MHC (PDB code 3t0e), and CTLA-4/CD80 (PDB code 1i8l) are shown. The approximate dimensions of the interacting protein complexes are shown. The last observed COOH-terminal residue in each structure is represented as a sphere. The number of amino acid (aa) residues that link the protein structures to their predicted transmembrane regions are given. Observed NAG and mannose moieties are shown as pink sticks and the sites of potential N-linked glycosylation sites are shown as pink spheres.

The structure of the CD200/CD200R complex allows the topology of the interaction between two opposing cells to be modeled. The distance between the last residues of each molecule in the structural model is ∼12 nm; this appears somewhat smaller than the 14 nm typically found for protein interactions spanning the immunological synapse; however, a further nine residues of CD200R need to be accounted for before entering the predicted transmembrane region. Allowing for these linking residues suggests that the distance spanned between two opposing cells is likely to be similar to other well-characterized interactions involved in fine tuning of the immune response such as those found in the T cell immunologic synapse (Figure 7). As this interaction occurs between mostly myeloid cells and a variety of other cell types including epithelial cells, the topology indicated supports a previous proposal (Wright et al., 2000) that synapse-like interactions may be found in a variety of cell types and more specifically to regulate myeloid cell function (such as CD200/CD200R and CD47/SIRPα; Figure 7).

There are many functional data indicating that the CD200/CD200R interaction acts between cells (trans) (Foster-Cuevas et al., 2004; Kretz-Rommel et al., 2007). The possibility that it occurs also in *cis* seems unlikely given the steric constraints from the extensive glycosylation of both proteins, in particular at residues Asn127 and Asn151 of CD200. In addition CD200 is widely expressed compared to CD200R and rarely on the same cells so this is unlikely to be a major factor (Mukhopadhyay et al., 2010; Wright et al., 2001).

### Implications of Paired Receptor Function and Evolution

#### The CD200/CD200R Interaction and Viral Orthologs

**Figure 8:**
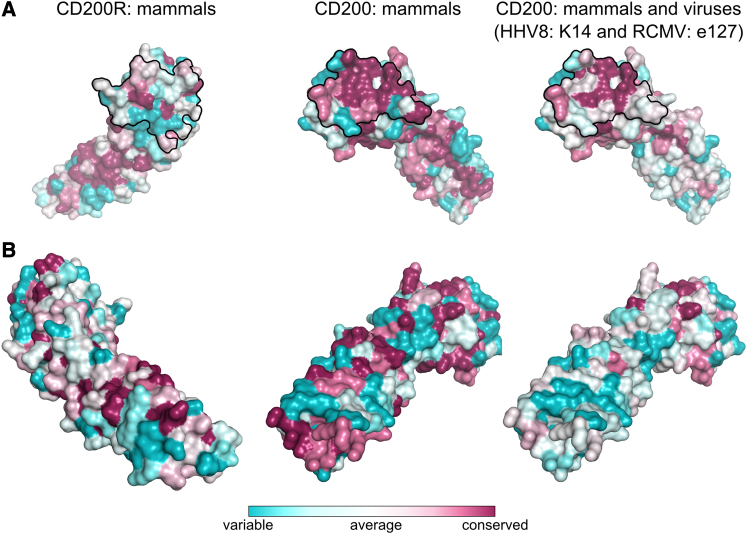
The CD200/CD200R Binding Site Is Conserved in Mammals and Some Viruses The ConSurf Server (Ashkenazy et al., 2010) was used to estimate the evolutionary conservation of amino acids in CD200 and CD200R using a multiple sequence alignment of mammalian (and two viral) CD200 and CD200R orthologs (generated by Clustal W2 (Larkin et al., 2007) and the CD200/CD200R coordinates. (A) Left panel: conservation of CD200R amino acids in the following mammals: mouse (NP_067300.1), human (NP_620161.1), chimp (XP_001156654.1), monkey (XP_001105494.1), wolf (XP_545099.2), cow (XP_002684796.2), and rat (NP_076443.1). The view of CD200R is the same as that in Figure 3C. Centere panel: conservation of CD200 amino acids in the following mammals: mouse (NP_034948.3), human (NP_001004196.2), chimp (XP_516648.3), monkey (XP_001104031.1), wolf (XP_849283.2), cow (NP_001029792.1), and rat (NP_113706.1). The view of CD200 is the same as that in Figure 3D. Right panel: conservation of CD200 amino acids in mammals (as in center panel) and the viral orthologs K14 (AAK53415.1) and e127 (AAO45420.1) from the human herpes virus 8 (HHV8) and the rat cytomegalovirus (RCMV), respectively. The black line outlines the residues at the CD200/CD200R interface. The relative degrees of residue conservation are colored from blue for variable positions to dark pink for conserved residues. (B) CD200R and CD200 are rotated 180° from (A).

When the sequences of CD200 and CD200R are compared across species, it is clear that CD200R is evolving more rapidly that CD200 (Figure 8). Viruses are constantly evolving to subvert the immune system and CD200-like orthologs containing two Ig-like domains have been identified in beta- and gammaherpesviridae (Foster-Cuevas et al., 2004). The human herpesvirus-8 (HHV-8; Kaposi sarcoma virus) CD200 ortholog (K14) mimics the interaction of human CD200 with CD200R to suppress macrophage and T cell responses to virus and K14 binds CD200R with the same affinity as the host CD200 protein (Foster-Cuevas et al., 2004; Misstear et al., 2012). The rat cytomegalovirus (RCMV) CD200 ortholog e127 also mimics the host protein in terms of affinity (Foster-Cuevas et al., 2011). Most of the residues in K14 and e127 predicted to be at the CD200R interface are mostly either identical or conservative changes compared to human and rat CD200, respectively, although the overall amino acid sequence identity in the V-like domain is 41% and 87%, respectively (Foster-Cuevas et al., 2004). This is illustrated in Figure 8, which compares the CD200s from mammals and includes the viral orthologs; it shows there is considerable divergence in domain 2 but less in the binding face in domain 1. It has been suggested that the heterogeneity in the paired receptors is a result of pathogen pressure where pathogens target the inhibitory receptors. These then evolve both by sequence change and by the generation of activating genes that no longer bind the host ligand but may provide decoys for pathogens targeting the inhibitory receptor (the counterbalance theory; Barclay and Hatherley, 2008). With regard to CD200/CD200R, the viral orthologs of CD200 may not be the driving force for the evolution of these activating receptors because they mimic the host CD200 so closely as indicated by their similar affinities. Thus the evolution of the CD200R family may have been driven by as yet unidentified pathogens.

### Conclusions

The structures indicate that CD200 and CD200R consist of two typical Ig-like domains interacting though their NH_2_-terminal domains so that the interaction has dimensions compatible with functioning in an immunological synapse. What is unusual about this interaction is that CD200 is expressed widely on endothelial and epithelial cells, suggesting that immunological style synapses may be important in these types of interactions in addition to the well-characterized interactions between leukocytes. Restricting the interaction to synapse-like regions may limit the conditions in which the receptor gets phosphorylated and may restrict access to pathogens targeting this receptor as suggested for the CD47/SIRPα interaction (Barclay and Hatherley, 2008; Hatherley et al., 2008). This interaction has many similarities to the CD200/CD200R interaction whereby a widely distributed ligand (CD47) reacts with a receptor restricted to myeloid cells (SIRPα) and the interaction is also a target for enhancing immunity to cancer (Chao et al., 2012).

One feature of CD200R is its extensive glycosylation (eight to ten N-linked sites depending on species) and that this glycosylation is distant from the interacting faces (Figure 3A). It is possible that this prevents unwanted interactions that might occur during the rapid evolution of the receptor and indeed this may be a common role for glycosylation of many leukocyte receptors that are generally evolving fast compared to those of other cell types (Barclay, 2003).

The finding that subtle changes around the binding site are responsible for the failure of the activating receptors to bind CD200 is reminiscent of other paired receptors such as the SIRPs (Hatherley et al., 2008). This suggested that the activating receptor has evolved from the inhibitory receptor and specifically to not bind host ligand. In this way it provides a defense mechanism for pathogens that are targeting the inhibitory receptors (discussed in Barclay and Hatherley, 2008). The finding that many virus types have acquired CD200 orthologs supports this concept. In some viruses (e.g., HHV8 and rCMV), the CD200 ortholog acts as a close mimic in terms of binding. Thus the structures provide evidence for a paired receptor system involved in homeostasis that is evolving rapidly under pathogen pressure. Given the importance of myeloid cells and their expression of CD200R, it seems likely that interactions with the microbiome may well be reflected in setting the levels of immune responses (Akkaya and Barclay, 2013). The presence of polymorphisms and heterogeneity in the repertoire of the CD200 paired receptor system may be important in disease susceptibility.

## Experimental Procedures

### Expression and Production of Recombinant Proteins for Crystallization

Recombinant mouse CD200RLa comprising the leader sequence, the two extracellular IgSF domains (residues 1–237, accession number NP_997127) followed by a C-terminal polyhistidine tag (STRHHHHHH) was expressed using the pEE14 vector in the CHO Lec3.2.8.1 cell line. The extracellular regions of mouse CD200R (residues 1–232; accession number NP_067300) and mouse CD200 (residues 1–232; accession number NP_034948) were also expressed with a C-terminal polyhistidine tag in the same manner as mouse CD200RLa.

The recombinant proteins were purified by nickel affinity chromatography and buffer exchanged into 10 mM HEPES pH 7.5, 150 mM NaCl, 0.02% NaN_3_ (HBS). N-linked sugars were removed using 2.5 U of Endoglycosidase Hf (NEB) per microgram of protein. Recombinant CD200R and CD200 were further purified by passing through a concanavalin A (Con A) Sepharose 4B column (GE Lifesciences), collecting the protein in the flow-through. Recombinant CD200 and CD200R eluted from the concanavalin A column were used as ligands in mutagenesis experiments (see subsequent section). All recombinant proteins were gel-filtrated into HBS buffer to remove contaminants and aggregated protein.

NH_2_-terminal protein sequences were determined using an automated Edman degradation in an Applied Biosystems Procise 494A protein sequencer (Perkin-Elmer Ltd, UK).

### Crystallization

Sitting drop vapor diffusion crystallization experiments were performed using an OryxNano robot (Douglas Instruments) to dispense nanoscale protein-precipitant drops that were equilibrated against precipitant reservoirs at either 12°C or 20°C. CD200RLa was concentrated to 9.6 mg/ml and P6_5_22 crystals grew (within 24 hr) from 300 nl drops containing 50% and 66% protein from 0.2 M sodium chloride, 0.1 M sodium cacodylate pH 6.5, and 2.0 M ammonium sulfate at 12°C. Crystals were cryoprotected in mother liquor supplemented with 20% glycerol and flash frozen in liquid nitrogen.

P4_1_2_1_2 crystals of CD200R grew from drops containing a 1:1 molar mix of CD200 and CD200R (each protein at 320 μM). Crystals grew (within 2 weeks) from 400 nl drops containing 50% and 70% protein from 0.2 M ammonium acetate, 0.1 M sodium acetate pH 4.0, 15% w/v PEG 4000 at 20°C. The CD200R crystal was cryoprotected in mother liquor supplemented with 20% glycerol and flash frozen in liquid nitrogen.

For the CD200/CD200R complex (each protein at 350 μM), crystals grew at 20°C from a 400 nl drop containing 70% protein from 1.0 M imidazole pH 7.0 and were cryoprotected in mother liquor containing 35% ethylene glycol and flash frozen in liquid nitrogen.

### Data Processing and Structure Determination

A native X-ray diffraction data set for mouse CD200RLa was collected at the European Synchrotron Radiation Facility (ESRF, Grenoble) at a wavelength of 0.9762 Å and was processed using Mosflm (Leslie, 1992) and SCALA (Evans, 1997). To obtain heavy metal derivatives, CD200RLa crystals were soaked in 65 mM gadolinium chloride for 2 minutes, cryoprotected in mother liquor containing 20% glycerol, and flash frozen in liquid nitrogen. A gadolinium derivative diffraction data set was collected at the Diamond Light Source (UK) at 1.7112 Å (the LIII absorption edge). Derivative data were processed using the Xia2 data processing suite (Winter, 2010). The autoSHARP suite of programs (Fortelle and Bricogne, 1997) was used to generate initial phases in a single isomorphous replacement with anomalous signal (SIRAS) experiment using the native and derivative data sets. SHELX D found six gadolinium sites and SHARP refined the coordinates, occupancy, and B factors for these sites and generated residual maps and phases. Solvent flattening was performed by SOLOMON to give new phases to 2.5 Å into which 561 residues of mCD200RLa were autobuilt by the Buccaneer program (Cowtan, 2006). Iterative cycles of manual model building in COOT (Emsley and Cowtan, 2004) and refinement with BUSTER-TNT (Blanc et al., 2004) were performed.

Diffraction data for CD200R and the CD200/CD200R complex were collected at Diamond on beamline I04 at 100 K (Table S1). Diffraction data sets were processed using Xia2 (Winter, 2010). The structure of CD200R was determined by molecular replacement using Phaser (McCoy et al., 2007) with the CD200RLa structure as a search model. Domains 1 and 2 of CD200RLa were initially used sequentially as molecular replacement search models by Phaser to determine initial phases for CD200R. Buccaneer was used to autobuild followed by iterative cycles of refinement with autoBUSTER (Bricogne et al., 2011) and manual model building in COOT.

The structure of the CD200/CD200R complex was initially solved by molecular replacement using Phaser with domains 1 and 2 of CD200R. Phases calculated from this partial model using autoBUSTER produced a map with clear density that allowed placement of a canonical immunoglobulin domain (domain 1 from 3ALP). Refinement of this model allowed manual placement of the second domain. Iterative cycles of refinement with autoBUSTER (Bricogne et al., 2011) and manual model building in COOT produced a complete model for CD200, with the positions of the glycosylated Asn residues verifying the remodeled sequence. Molecular graphics were generated using Pymol (http://www.pymol.org; Schrödinger). Electrostatic surface potentials were calculated using the Pymol APBS Tools 2 plugin. The CD200/CD200R structure was analyzed using the protein interfaces, surfaces, and assemblies service PISA at European Bioinformatics Institute (http://www.ebi.ac.uk/pdbe/prot_int/pistart.html) (Krissinel and Henrick, 2007).

### CD200 Mutagenesis and Analysis by Surface Plasmon Resonance

Surface plasmon resonance experiments were performed using a BIAcore 3000 at 25°C. Recombinant CD200R and CD200 eluted from respective Con A columns (see expression section above) were gel-filtrated in HBS. The extracellular region of CD200 (residues 1–232) was tagged at the C terminus with domains 3 and 4 of rat CD4 (CD4) followed by a biotinylation (Bio) site and transiently expressed by 293T cells as described previously (Brown et al., 1998). Single point mutants of CD200 were generated by overlap extension polymerase chain reaction. OX68 mAb (mouse anti-rat CD4) was amine coupled to a CM5 chip and approximately 650 response units (RUs) of each protein immobilized. Serial dilutions of CD200R were injected over immobilized wild-type and mutant CD200 and CD4 control protein. A k_D_ of 0.5 μM was calculated from equilibrium binding data and this concentration of CD200R was used to test the binding ability of mutant CD200 proteins compared to wild-type protein. A CD200 mAb (OX90) was passed over the proteins at 0.2 mg/ml. The ligand and antibody responses obtained for the control CD4 protein were subtracted from the wild-type and mutant CD200 responses to obtain the specific binding response for each protein. To compensate for any variation in protein levels immobilized on each flow cell the specific binding response for each protein was divided by the amount of protein immobilized and then expressed as a percentage of wild-type protein (100%). The OX68 chip was regenerated by passing over 0.1 M glycine HCl pH 2.0 to remove immobilized proteins.

Similarly, CD200R (residues 1–232) and wild-type and mutant CD200RLa (residues 1–237) CD4-Bio chimeric proteins were expressed and immobilized via OX68 onto a CM5 BIAcore chip. Increasing concentrations of CD200 were injected over the immobilized proteins and K_D_s were obtained by nonlinear curve fitting to the data.

## Accession Numbers

The atomic coordinates and structure factors for CD200R, CD200RLa, and the CD200R/CD200 complex reported in this paper have been deposited in the Protein Data Bank under ID codes 4bfg, 4bfe, and 4bfi, respectively.

## References

[bib1] Akkaya M., Barclay A.N. (2010). Heterogeneity in the CD200R paired receptor family. Immunogenetics.

[bib2] Akkaya M., Barclay A.N. (2013). How do pathogens drive the evolution of paired receptors?. Eur. J. Immunol..

[bib3] Ashkenazy H., Erez E., Martz E., Pupko T., Ben-Tal N. (2010). ConSurf 2010: calculating evolutionary conservation in sequence and structure of proteins and nucleic acids. Nucleic Acids Res..

[bib4] Barclay A.N. (2003). Membrane proteins with immunoglobulin-like domains—a master superfamily of interaction molecules. Semin. Immunol..

[bib5] Barclay A.N., Ward H.A. (1982). Purification and chemical characterisation of membrane glycoproteins from rat thymocytes and brain, recognised by monoclonal antibody MRC OX 2. Eur. J. Biochem..

[bib6] Barclay A.N., Brown M.H. (2006). The SIRP family of receptors and immune regulation. Nat. Rev. Immunol..

[bib7] Barclay A.N., Hatherley D. (2008). The counterbalance theory for evolution and function of paired receptors. Immunity.

[bib8] Barclay A.N., Wright G.J., Brooke G., Brown M.H. (2002). CD200 and membrane protein interactions in the control of myeloid cells. Trends Immunol..

[bib9] Blanc E., Roversi P., Vonrhein C., Flensburg C., Lea S.M., Bricogne G. (2004). Refinement of severely incomplete structures with maximum likelihood in BUSTER-TNT. Acta Crystallogr. D Biol. Crystallogr..

[bib10] Bricogne G., Blanc E., Brandl M., Flensburg C., Keller P., Paciorek W., Roversi P., Sharff A., Smart O.S., Vonrhein C., Womack T.O. (2011). BUSTER version 2.11.2.

[bib11] Brown M.H., Boles K., van der Merwe P.A., Kumar V., Mathew P.A., Barclay A.N. (1998). 2B4, the natural killer and T cell immunoglobulin superfamily surface protein, is a ligand for CD48. J. Exp. Med..

[bib12] Chao M.P., Weissman I.L., Majeti R. (2012). The CD47-SIRPα pathway in cancer immune evasion and potential therapeutic implications. Curr. Opin. Immunol..

[bib13] Clark M.J., Gagnon J., Williams A.F., Barclay A.N. (1985). MRC OX-2 antigen: a lymphoid/neuronal membrane glycoprotein with a structure like a single immunoglobulin light chain. EMBO J..

[bib14] Cowtan K. (2006). The Buccaneer software for automated model building. 1. Tracing protein chains. Acta Crystallogr. D Biol. Crystallogr..

[bib15] Davis S.J., Puklavec M.J., Ashford D.A., Harlos K., Jones E.Y., Stuart D.I., Williams A.F. (1993). Expression of soluble recombinant glycoproteins with predefined glycosylation: application to the crystallization of the T-cell glycoprotein CD2. Protein Eng..

[bib16] Fortelle E., Bricogne G., Carter C.W., Sweet R.M. (1997). Maximum-likelihood heavy-atom parameter refinement for multiple isomorphous replacement and multiwavelength anomalous diffraction methods. Methods in Enzymology.

[bib17] Emsley P., Cowtan K. (2004). Coot: model-building tools for molecular graphics. Acta Crystallogr. D Biol. Crystallogr..

[bib18] Evans P.R. (1997). Scala. Joint CCP4 + ESF-EAMCB Newsletter on Protein Crystallography.

[bib19] Foster-Cuevas M., Wright G.J., Puklavec M.J., Brown M.H., Barclay A.N. (2004). Human herpesvirus 8 K14 protein mimics CD200 in down-regulating macrophage activation through CD200 receptor. J. Virol..

[bib20] Foster-Cuevas M., Westerholt T., Ahmed M., Brown M.H., Barclay A.N., Voigt S. (2011). Cytomegalovirus e127 protein interacts with the inhibitory CD200 receptor. J. Virol..

[bib21] Gorczynski R.M., Cattral M.S., Chen Z., Hu J., Lei J., Min W.P., Yu G., Ni J. (1999). An immunoadhesin incorporating the molecule OX-2 is a potent immunosuppressant that prolongs allo- and xenograft survival. J. Immunol..

[bib22] Gorczynski R.M., Chen Z., Yu K., Hu J. (2001). CD200 immunoadhesin suppresses collagen-induced arthritis in mice. Clin. Immunol..

[bib23] Gorczynski R., Chen Z., Kai Y., Lee L., Wong S., Marsden P.A. (2004). CD200 is a ligand for all members of the CD200R family of immunoregulatory molecules. J. Immunol..

[bib24] Hatherley D., Barclay A.N. (2004). The CD200 and CD200 receptor cell surface proteins interact through their N-terminal immunoglobulin-like domains. Eur. J. Immunol..

[bib25] Hatherley D., Cherwinski H.M., Moshref M., Barclay A.N. (2005). Recombinant CD200 protein does not bind activating proteins closely related to CD200 receptor. J. Immunol..

[bib26] Hatherley D., Graham S.C., Turner J., Harlos K., Stuart D.I., Barclay A.N. (2008). Paired receptor specificity explained by structures of signal regulatory proteins alone and complexed with CD47. Mol. Cell.

[bib27] Kretz-Rommel A., Qin F., Dakappagari N., Ravey E.P., McWhirter J., Oltean D., Frederickson S., Maruyama T., Wild M.A., Nolan M.-J. (2007). CD200 expression on tumor cells suppresses antitumor immunity: new approaches to cancer immunotherapy. J. Immunol..

[bib28] Krissinel E., Henrick K. (2007). Inference of macromolecular assemblies from crystalline state. J. Mol. Biol..

[bib29] Larkin M.A., Blackshields G., Brown N.P., Chenna R., McGettigan P.A., McWilliam H., Valentin F., Wallace I.M., Wilm A., Lopez R. (2007). Clustal W and Clustal X version 2.0. Bioinformatics.

[bib30] Leslie A. (1992). Recent changes to the MOSFLM package for processing film and image plate data. Joint CCP4 + ESF-EAMCB Newsletter on Protein Crystallography.

[bib31] Mallett S., Fossum S., Barclay A.N. (1990). Characterization of the MRC OX40 antigen of activated CD4 positive T lymphocytes—a molecule related to nerve growth factor receptor. EMBO J..

[bib32] McCoy A.J., Grosse-Kunstleve R.W., Adams P.D., Winn M.D., Storoni L.C., Read R.J. (2007). Phaser crystallographic software. J. Appl. Cryst..

[bib33] Metcalfe C., Cresswell P., Ciaccia L., Thomas B., Barclay A.N. (2011). Labile disulfide bonds are common at the leucocyte cell surface. Open Biology.

[bib34] Mihrshahi R., Barclay A.N., Brown M.H. (2009). Essential roles for Dok2 and RasGAP in CD200 receptor-mediated regulation of human myeloid cells. J. Immunol..

[bib35] Misstear K., Chanas S.A., Rezaee S.A.R., Colman R., Quinn L.L., Long H.M., Goodyear O., Lord J.M., Hislop A.D., Blackbourn D.J. (2012). Suppression of antigen-specific T cell responses by the Kaposi’s sarcoma-associated herpesvirus viral OX2 protein and its cellular orthologue, CD200. J. Virol..

[bib36] Moreaux J., Hose D., Reme T., Jourdan E., Hundemer M., Legouffe E., Moine P., Bourin P., Moos M., Corre J. (2006). CD200 is a new prognostic factor in multiple myeloma. Blood.

[bib37] Mukhopadhyay S., Plüddemann A., Hoe J.C., Williams K.J., Varin A., Makepeace K., Aknin M.-L., Bowdish D.M.E., Smale S.T., Barclay A.N., Gordon S. (2010). Immune inhibitory ligand CD200 induction by TLRs and NLRs limits macrophage activation to protect the host from meningococcal septicemia. Cell Host Microbe.

[bib38] Nathan C., Muller W.A. (2001). Putting the brakes on innate immunity: a regulatory role for CD200?. Nat. Immunol..

[bib39] Rosenblum M.D., Olasz E.B., Yancey K.B., Woodliff J.E., Lazarova Z., Gerber K.A., Truitt R.L. (2004). Expression of CD200 on epithelial cells of the murine hair follicle: a role in tissue-specific immune tolerance?. J. Invest. Dermatol..

[bib40] Snelgrove R.J., Goulding J., Didierlaurent A.M., Lyonga D., Vekaria S., Edwards L., Gwyer E., Sedgwick J.D., Barclay A.N., Hussell T. (2008). A critical function for CD200 in lung immune homeostasis and the severity of influenza infection. Nat. Immunol..

[bib41] Stanley P. (1989). Chinese hamster ovary cell mutants with multiple glycosylation defects for production of glycoproteins with minimal carbohydrate heterogeneity. Mol. Cell. Biol..

[bib42] Tonks A., Hills R., White P., Rosie B., Mills K.I., Burnett A.K., Darley R.L. (2007). CD200 as a prognostic factor in acute myeloid leukaemia. Leukemia.

[bib43] Voehringer D., Rosen D.B., Lanier L.L., Locksley R.M. (2004). CD200 receptor family members represent novel DAP12-associated activating receptors on basophils and mast cells. J. Biol. Chem..

[bib44] Winter G. (2010). xia2: an expert system for macromolecular crystallography data reduction. J. Appl. Cryst..

[bib45] Wright G.J., Puklavec M.J., Willis A.C., Hoek R.M., Sedgwick J.D., Brown M.H., Barclay A.N. (2000). Lymphoid/neuronal cell surface OX2 glycoprotein recognizes a novel receptor on macrophages implicated in the control of their function. Immunity.

[bib46] Wright G.J., Jones M., Puklavec M.J., Brown M.H., Barclay A.N. (2001). The unusual distribution of the neuronal/lymphoid cell surface CD200 (OX2) glycoprotein is conserved in humans. Immunology.

[bib47] Wright G.J., Cherwinski H., Foster-Cuevas M., Brooke G., Puklavec M.J., Bigler M., Song Y., Jenmalm M., Gorman D., McClanahan T. (2003). Characterization of the CD200 receptor family in mice and humans and their interactions with CD200. J. Immunol..

[bib48] Zhang S., Cherwinski H., Sedgwick J.D., Phillips J.H. (2004). Molecular mechanisms of CD200 inhibition of mast cell activation. J. Immunol..

[bib49] Zhang L., Stanford M., Liu J., Barrett C., Jiang L., Barclay A.N., McFadden G. (2009). Inhibition of macrophage activation by the myxoma virus M141 protein (vCD200). J. Virol..

