# Supplementary material for: Structures of CD200/CD200 Receptor Family and Implications for Topology, Regulation, and Evolution
Source: Structure. 2013 May 7;21(5):820–32. doi: 10.1016/j.str.2013.03.008 (PMC3664923; doi:10.1016/j.str.2013.03.008)
Supplement: Document S1. Figures S1–S4 and Table S1 [file mmc1.pdf]

Structure, Volume *21*

Supplemental Information

Structures of CD200/CD200 Receptor Family and Implications for Topology, Regulation, and Evolution

Deborah Hatherley, Susan M. Lea, Steven Johnson, and A. Neil Barclay

Inventory of Supplemental Information

Figure 1. This describes the purified proteins and the extent of the removal of the carbohydrate that is a major part of the glycoprotein, Related to Experimental Procedures

Figure 2. This indicates two points – one an indication of the fit of the electron density data and secondly analysis of the density around the 'exposed Cys' as discussed in the text; these are the raw data, Related to Figure 2

Figure 3. The movement in CD200R on binding CD200 is discussed in the text. This figure makes it much easier to visualize what is happening, Related to Figure 3

Figure 4. This is the alignment of the activating members of the CD200R family and allows discussion of the changes that lead to no binding, Related to Figure 6

Table 1. This contains the raw data on the mutagenesis. The results can be illustrated in the figures in the text but these data show the detailed quantitative analysis that has gone into working out the contributions of residues to the gain of function, Related to Figure 5

Supplementary Figures

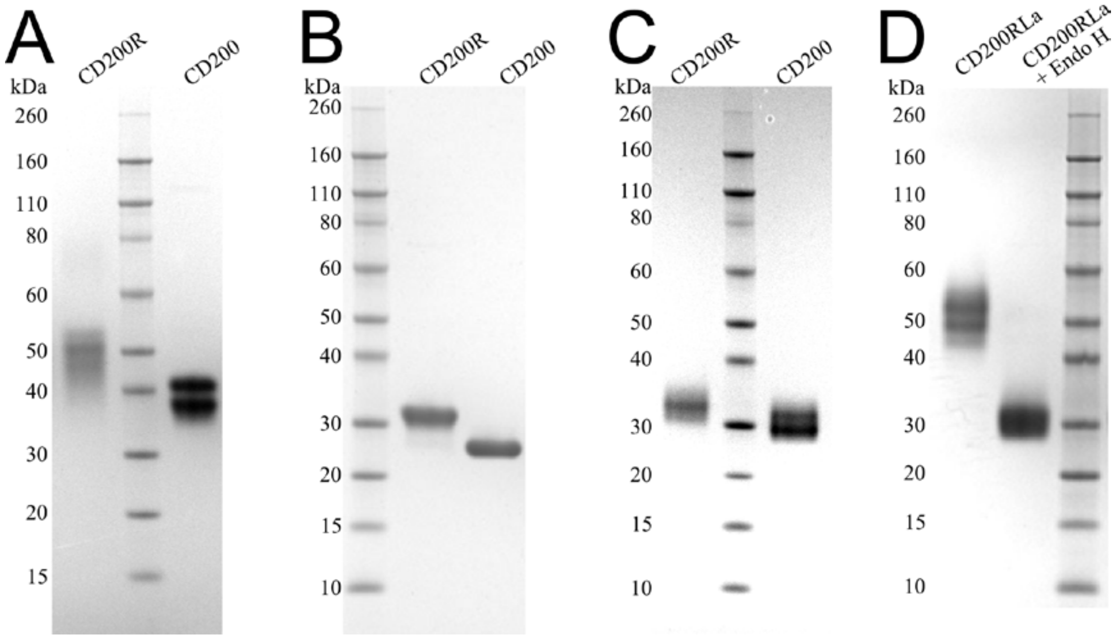

Figure S1. Purified proteins used for X-ray crystallography. SDS PAGE analysis shows deglycosylation of recombinant proteins. (A) Ni-NTA purified CD200R and CD200 produced by CHO Lec3.2.8.1 cells. (B) Deglycosylated CD200R and CD200 used for crystallisation are shown. Proteins were deglycosylated using endo H_f, passed through a concanavalin A Sepharose™ column and gel filtrated. (C) Endo H_f resistant glycoforms of CD200 and CD200R were eluted from a concanavalin sepharose column, gel filtrated and used for ligand binding experiments using a BIAcore. (D) Glycosylated CD200RLa produced in CHO Lec3.2.8.1 cells (first lane) and deglycosylated CD200RLa used for crystallisation (second lane) are shown.

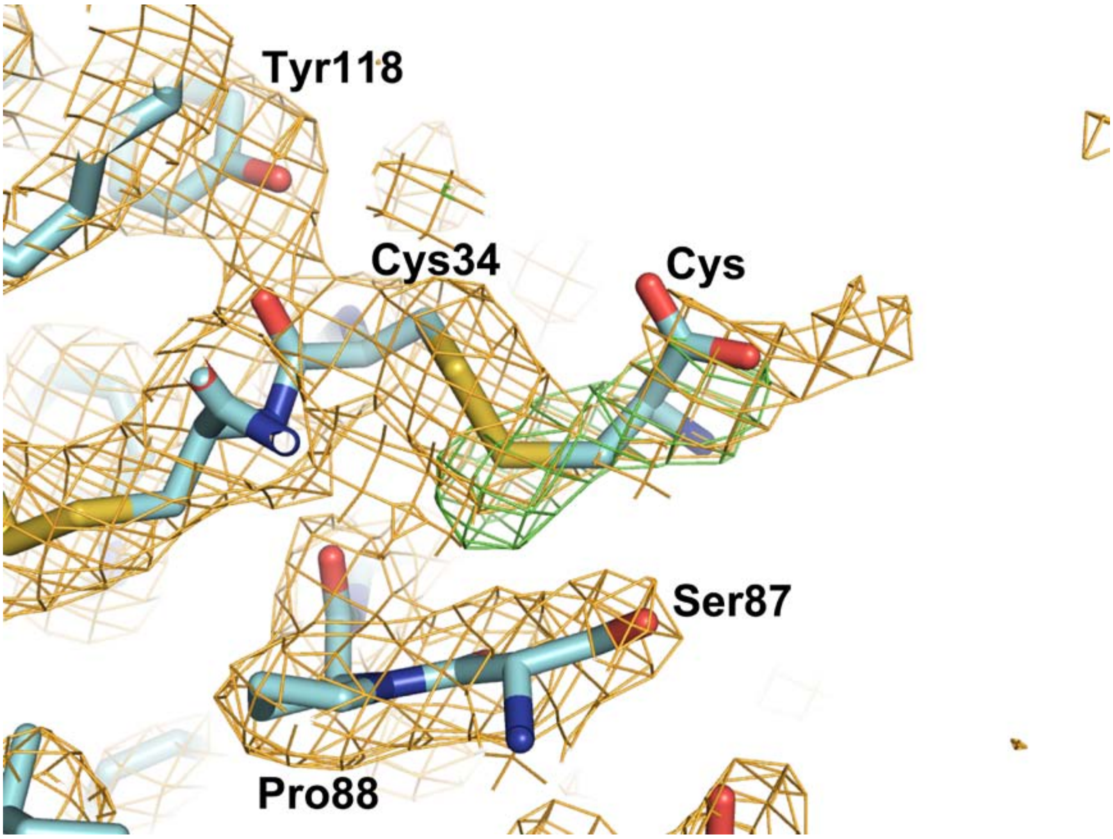

Figure S2, related to Figure 2. Electron density showing the extra density attached to Cys34 in copy A of mCD200RLa. 2Fo-Fc (orange, 1sigma) and Fo-Fc (green, 3sigma) maps were calculated before anything was modelled in the density. Also shown are the final refined coordinates with a Cys residue bonded to Cys34.

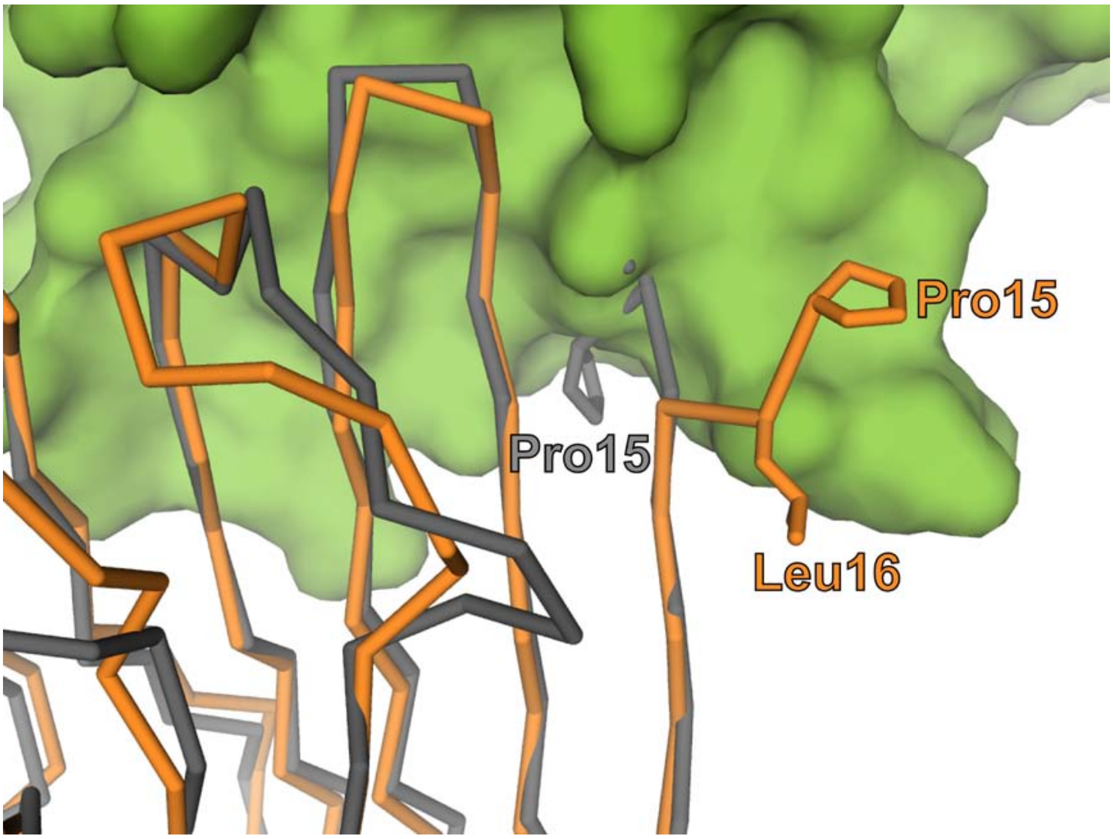

Figure S3, related to Figure 3. CD200 (green surface) shown complexed with CD200R (orange ribbon) and a virtual complex with free CD200R (grey ribbon). Pro15 and Leu16 move from the GFC face to the BED face due to an approximately 120 degree rotation of the psi angle of Thr17, thereby exposing the full CD200 binding site.

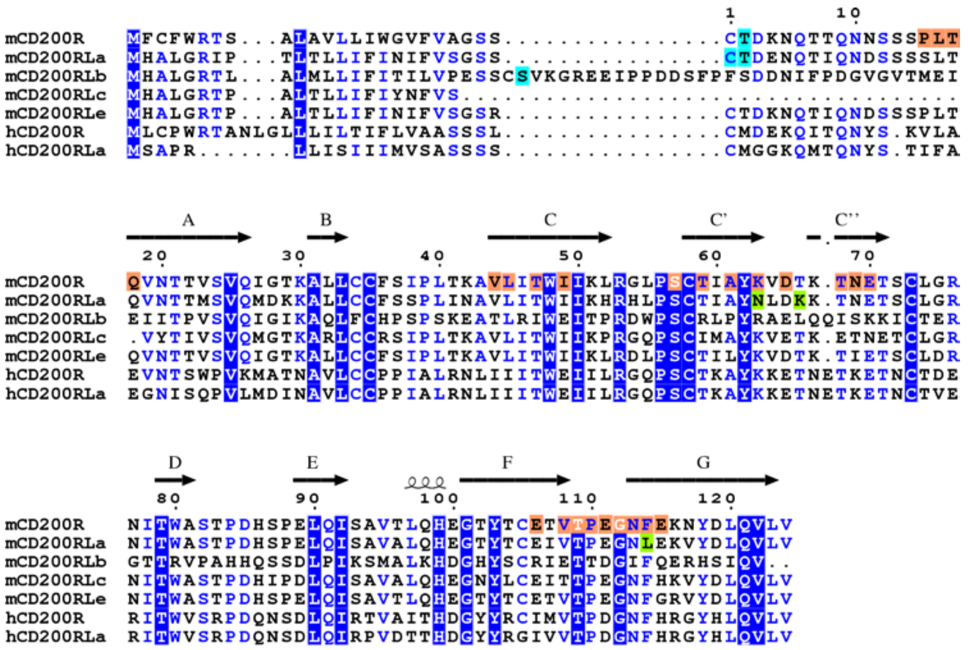

Figure S4, related to Figure 6. Sequence alignment of the leader and V-like domain of the CD200R family. Amino acid secondary structure is based on the mouse CD200R crystal structure with beta strands represented by arrows. Conserved residues are highlighted in blue and residues identical in five or more sequences are in blue type. NH_2-terminal residues determined by protein sequencing are highlighted in cyan. mCD200R residues at the CD200 / CD200R interface are highlighted in orange. The residues of mCD200RLa that upon mutation resulted in binding to CD200 (with a similar affinity as mCD200R) are highlighted in green. Accession numbers of the sequences are NP_067300 (mCD200R), NP_997127 (mCD200RLa), NP_001121604 (mCD200RLb), Q6XJV6 (mCD200RLc), Q8BTP3 (mCD200RLe), NP_740750 (hCD200R), and NP_001008784 (hCD200RLa).

Table S1, related to Figure 5. The ability of CD200 mutants to bind CD200R compared to wild type CD200 determined by BIAcore analysis as in Figure 5. The experiment was repeated twice and the average result is given.

	CD200	CD200R protein binding (%)
Residue location	Wild type	100
BC loop	L30K	3
C strand	I31E	0
C’ strand	N44D	1
C’ strand	N44A	2
F strand	L92A	11
F strand	N94K	1
FG loop	F96D	1
FG loop	F96A	3
FG loop	K100E	2
A strand	Q7K	102
B strand	S18D	95
BC loop	Q27K	145
